# Peptide receptor radiotherapy: a new option for the management of aggressive fibromatosis on behalf of the Italian Sarcoma Group

**DOI:** 10.1038/sj.bjc.6600823

**Published:** 2003-03-04

**Authors:** T De Pas, L Bodei, G Pelosi, F de Braud, G Villa, R Capanna, G Paganelli

**Affiliations:** 1 Division of Medical Oncology, European Institute of Oncology, Via Ripamonti 435-I-20141, Milan, Italy; 2 Division of Nuclear Medicine, European Institute of Oncology, Via Ripamonti 435-I-20141, Milan, Italy; 3 Pathology and Laboratory Medicine, European Institute of Oncology, Via Ripamonti 435-I-20141, Milan, Italy; 4 Division of Radiology, European Institute of Oncology, Via Ripamonti 435-I-20141, Milan, Italy; 5 2nd Division of Orthopedics and Traumatology, CTO, Florence, Italy

**Keywords:** fibromatosis, external-beam radiation therapy, receptor radiotherapy, desmoid tumour

## Abstract

The management of aggressive fibromatosis (AF) is problematic, and few options are available to patients unsuitable for surgery and resistant to external-beam radiation therapy (EBRT). We report on two patients with fast-growing recurrences of AF resistant to EBRT who obtained protracted clinical benefits with ^90^Y-DOTATOC. ^90^Y-DOTATOC should be further investigated in this setting.

Peptide receptor radiotherapy, which uses radiolabelled peptides that recognise tumour cell surface receptors, is a relatively new technique for delivering large quantities of radioactivity to tumours. The somatostatin analogue [DOTA^0^, Tyr^3^]octreotide (DOTATOC) has been shown to be useful for this purpose, as it is easily and stably labelled with yttrium-90. It has high affinity for the somatostatin receptor subtype 2 (sst2) and a favourable bio-distribution and safety profile in patients (Kwekkeboom *et al*, 2000). Tumour uptake of ^90^Y-DOTATOC can be revealed before treatment by scintigraphy with ^111^In-labelled octreotide (OctreoScan) (Krenning *et al*, 1993). When ^90^Y-DOTATOC is given to patients with cancers that express somatostatin receptors and show adequate OctreoScan uptake, a good tumour–response rate is observed (Cremonesi *et al*, 1999; Waldherr *et al*, 2001; Chinol *et al*, 2002).

Aggressive fibromatosis (AF) is a rare fibrous neoplasm with biological behaviour intermediate between that of benign fibrous lesions and malignant fibrosarcomas. The tumour is poorly circumscribed and grows along tissue planes with an infiltrating-like pattern towards mesenchymal tissue. The clinical equivalent to this pathological behaviour is a high tendency to local recurrence. Its management is problematic: adequate surgery is the first-choice treatment; however, the lesion tends to recur locally even if surgically removed with pathologically documented free-margin resection. As systemic treatment options, chemotherapy, hormone therapy and NSAIDs have been used with mixed success.

The response of AF to external-beam radiation (EBRT) is documented, with a dose–response relation for tumour doses of less than 50 Gy (Greenberg *et al*, 1981; Nuyttens *et al*, 2000). However, there are AF cases that cannot be managed with EBRT because of normal-tissue constraints, as there are cases that appear to be resistant or refractory to EBRT.

According to these data, and to a report of partial AF regression after prolonged treatment with the somatostatin-analogue lanreotide (Mannant *et al*, 1995), we decided to investigate the usefulness of ^90^Y-DOTATOC as a treatment for recurrent AF.

## MATERIALS AND METHODS

Aggressive fibromatosis specimens from 10 patients were retrospectively examined for immunopositivity to a rabbit polyclonal antibody anti-sst2A (cod SS-800, Biotrend Chemikalien, Germany). Two specimens showed immunoreactivity to sst2. Next, we used OctreoScan in six other patients with AF to test the expression of sst2 receptors on the neoplasm.

Two patients had AF lesions that showed adequate OctreoScan uptake (i.e., at least equal to one of the liver) and were hence eligible for treatment with ^90^Y-DOTATOC. We explained to these two patients the potential risks and benefits of this experimental treatment, which had been approved by our Institute's ethical review board. They gave their written consent. An ongoing phase I–II study using ^90^Y-DOTATOC in patients with solid tumours (Paganelli *et al*, 2001; Chinol *et al*, 2002; Bodei *et al*, 2003) was followed for these patients.

According to this protocol, patients had histologically confirmed cancers expressing sst_2_ receptors, as documented by OctreoScan, and documented residual disease or recurrence after conventional treatment. DOTATOC was synthesised at the Division of Radiological Chemistry, University Hospital, Basel using a published procedure (Heppeler *et al*, 1999). Yttrium-90 chloride was purchased from AEA Technology (Harwell, UK). DOTATOC molecule was radiolabelled according to a published procedure (Chinol *et al*, 2002). ^90^Y-DOTATOC was injected intravenously over 20 min in 100 ml of physiological saline. Patients received renal protection by infusing 10 g lysine hydrochloride in 500 ml of saline over an hour before therapy and 15 g of lysine hydrochloride in 750 ml saline over 2 h after therapy. The patients were hospitalised for 2–3 days after treatment in rooms set aside for radionuclide therapy, and were discharged only after the level of specific activity in the urine had fallen below 0.037 MBq ml^−1^, as stipulated in the regulations governing use of radionuclides at our centre.

Toxicity was evaluated according to WHO criteria (Miller *et al*, 1981). After discharge from hospital the patients underwent the following tests: renal function, hepatic function, LDH and uricaemia every 30 days, complete blood count every 15 days for the first 2 months and monthly subsequently.

## RESULTS

### Case 1

**Figure 1:**
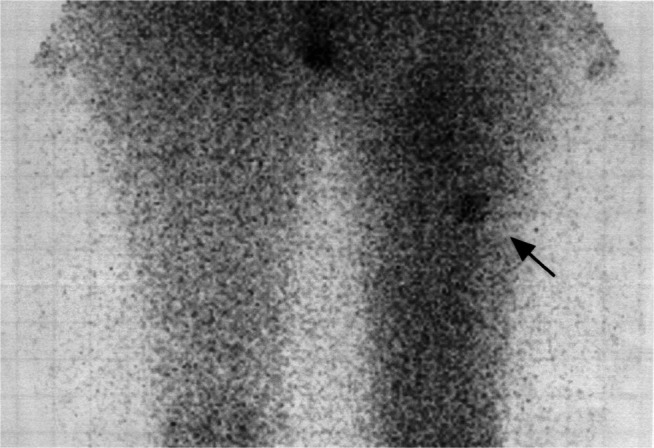
The patient had a local relapse of right-thigh aggressive fibromatosis: a scintigraphy with ^111^In-labelled octreotide (OctreoScan) revealed tumour uptake of the radiopeptide (arrow).

The first patient, a 30-year-old woman, had a local relapse of right-thigh AF. Nine months after the primary tumour had been radically removed, she underwent further surgery for a local relapse; however, during subsequent adjuvant external-beam radiotherapy the disease again recurred and grew rapidly. The patient received i.v., ^90^Y-DOTATOC in five cycles over 14 months, cumulative activity 10.6 GBq. There was a partial response on MRI according to WHO criteria after the first cycle (2.22 GBq) which has lasted for more than 37 months (Figures 1

and 2

**Figure 2:**
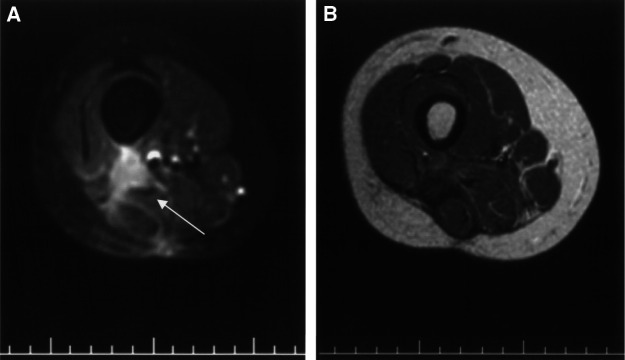
(**A**) Magnetic resonance (SE FS T1W post Gd-DTPA Image) showed a local relapse of right-thigh aggressive fibromatosis (arrow). (**B**) A partial response of disease (WHO criteria) was obtained by treating with ^90^Y-DOTATOC (SE T1W post Gd-DTPA magnetic resonance image).

).

### Case 2

The second patient was a 22-year-old man with AF of the right thigh. After radical surgery and adjuvant radiotherapy (44 Gy external-beam and 27 Gy brachytherapy), there were four relapses in the following 4 years. The relapses were removed surgically and postoperative external-beam reirradiation (35 Gy) was given after the third operation.

For the fifth relapse, characterised by rapid growth, conservative surgery and radiotherapy were no longer possible, and as an alternative to amputation we administered a cumulative activity of 8.18 GBq of ^90^Y-DOTATOC in three cycles over 4 months. There was a disease stabilisation on MRI according to WHO criteria after the first cycle (3.26 GBq) and disease has remained stable for more than 19 months.

^90^Y-DOTATOC was very well tolerated in these patients, and neither acute nor late toxicity was observed.

## DISCUSSION

Patients with recurrent AF after full exploitation of the standard therapy modalities such as surgery and EBRT are lacking other local therapeutic options. In this communication, we report on two patients with fast-growing recurrence of AF that appeared to be resistant to appropriate EBRT.

Both patients obtained a protracted clinical benefit with a peptide receptor radiotherapy with ^90^Y-DOTATOC in the absence of acute and late toxicity. The excellent tolerability of this experimental treatment is consistent with the results of our phase I–II study (Paganelli *et al*, 2001; Chinol *et al*, 2002; Bodei *et al*, in press). In this study, toxicity due to ^90^Y-DOTATOC, which was mainly haematological, occurred in a minority of patients receiving high activities per cycle (grade III toxicity in three out of seven — 43% — of the patients receiving 5.18 GBq, defined as MTD/cycle) and tended to reverse within 4 weeks. Neither permanent renal toxicity nor other forms of toxicity were observed.

To our knowledge, this is the first report showing that ^90^Y-DOTATOC can provide clinical benefits in patients with AF, and the benefit obtained supports further investigation in this setting. An OctreoScan should first be performed on a candidate patient with AF and if adequate tumour uptake is observed, ^90^Y-DOTATOC could be considered as a further treatment option to increase the chance of obtaining disease control.

## References

[bib1] Bodei L, Cremonesi M, Zoboli S, Grana C, Bartolomei M, Rocca P, Caracciolo M, Mäcke HR, Chinol M, Paganelli G (2003) Receptor-mediated radionuclide therapy with ^90^Y-DOTATOC in association with amino acid infusion: a phase I study. Eur J Nucl Med. Published online: 16 November 2002 10.1007/s00259-002-1023-y 12552338

[bib2] Chinol M, Bodei L, Cremonesi M, Paganelli G (2002) Receptor-mediated radiotherapy with ^90^Y-DOTA-D-Phe^1^-Tyr^3^-octreotide: the experience of the European Institute of Oncology group. Semin Nucl Med 32: 141–147 11965609 10.1053/snuc.2002.31563

[bib3] Cremonesi M, Ferrari M, Zoboli S, Chinol M, Stabin MG, Orsi F, Maecke HR, Jermann E, Robertson C, Fiorenza M, Tosi G, Paganelli G (1999) Biokinetics and dosimetry in patients administered with (111)In-DOTA-Tyr(3)-octreotide: implications for internal radiotherapy with (90)Y-DOTATOC. Eur J Nucl Med 26: 877–886 10436201 10.1007/s002590050462

[bib4] Greenberg HM, Goebel R, Weichselbaum RR, Greenberger JS, Chaffey JT, Cassady JR (1981) Radiation therapy in the treatment of aggressive fibromatosis. Int J Radiat Oncol 7: 305–310 10.1016/0360-3016(81)90102-4 6792167

[bib5] Heppeler A, Froidevaux S, Mäcke HR, Jermann E, Béhé M, Powell P, Hennig M (1999) Radiometal-labelled macrocyclic chelator–derivatised somatostatin analogue with superb tumour-targeting properties and potential for receptor–mediated internal radiotherapy. Chem Eur 7: 1974–1981

[bib6] Krenning EP, Kwekkeboom DJ, Bakker WH, Breeman WAP, Kooij PPM, Oei HY, Vanhagen M, Postema PTE, Dejong M, Reubi JC, Visser TJ, Reis AEM, Hofland LJ, Koper JW, Lamberts SWJ (1993) Somatostatin receptor scintigraphy with [^111^In-DTPA-D-Phe^1^]-and [^123^I-Tyr^3^]-octreotide: the Rotterdam experience with more than 1000 patients. Eur J Nucl Med 20: 716–731 8404961 10.1007/BF00181765

[bib7] Kwekkeboom D, Krenning EP, de Jong M (2000) Peptide receptor imaging and therapy. J Nucl Med 41: 1704–1713 11038002

[bib8] Mannant PR, Beau P, De Ledinghen V, Barrioz T, Genestin E (1995) Partial regression of a desmoid tumor after prolonged treatment with lanreotide. Gastroenterol Clin Biol 19: 1072–1073 8729428

[bib9] Miller AB, Hoogstraten B, Staquet M, Winkler A (1981) Reporting results of cancer treatment. Cancer 47: 207–214 7459811 10.1002/1097-0142(19810101)47:1<207::aid-cncr2820470134>3.0.co;2-6

[bib10] Nuyttens JJ, Rust PF, Thomas Jr CR, Turrisi III AT (2000) Surgery versus radiation therapy for patients with aggressive fibromatosis or desmoid tumors: a comparative review of 22 articles. Cancer 88: 1517–1523 10738207

[bib11] Paganelli G, Zoboli S, Cremonesi M, Bodei L, Ferrari M, Grana C, Bartolomei M, Orsi F, De Cicco C, Macke HR, Chinol M, de Braud F (2001) Receptor-mediated radiotherapy with ^90^Y-DOTA-D-Phe^1^-Tyr^3^-octreotide. Eur J Nucl Med 28: 426–434 11357492 10.1007/s002590100490

[bib12] Waldherr C, Pless M, Maecke HR, Haldemann A, Mueller-Brand J (2001) The clinical value of [90Y-DOTA]-D-Phe1-Tyr3-octreotide (^90^Y-DOTATOC) in the treatment of neuroendocrine tumors: a clinical phase II study. Ann Oncol 12: 941–945 11521799 10.1023/a:1011160913619

